# 1335. Retrospective evaluation of doxycycline for the treatment of Lyme disease among young children

**DOI:** 10.1093/ofid/ofac492.1165

**Published:** 2022-12-15

**Authors:** Katie Brown, Andrew S Handel

**Affiliations:** Stony Brook Children's Hospital, Stony Brook, New York; Stony Brook Children's Hospital, Stony Brook, New York

## Abstract

**Background:**

Lyme disease is the most common pediatric tickborne infection. Due to largely disproven concerns of doxycycline-related dental staining among patients < 8 years old, no prospective data exist on efficacy and adverse effects in young children with Lyme disease. Based on emerging data, our institution has used doxycycline during the past decade when alternatives cannot be given. Our study aims to describe short-term adverse effects and treatment failures among young children receiving oral doxycycline for Lyme disease.

**Methods:**

Retrospective chart review of patients < 8 years old within the Stony Brook Medicine system from 2010-2020. Patients were identified by ICD-9/-10 codes for Lyme disease prescribed doxycycline at the same visit. We excluded those lacking an objective finding of Lyme disease, including single or multiple erythema migrans (EM), facial nerve palsy, carditis, meningitis (headache with pleocytosis >5 WBC/μL), and/or arthritis, those with an alternative diagnosis, and those receiving post-tick exposure prophylactic doxycycline. Data collected included demographics, symptoms, laboratory results, treatments, and outcomes. Descriptive statistics were calculated.

**Results:**

32 charts were included. Average age was 5.1 years. 66% were male. The majority of the patients presented with a single EM (Table for presenting symptoms). Initial antibiotics were doxycycline (63%) or a beta-lactam (37%). Rationale for doxycycline included beta-lactam allergy or intolerance (47%), required for treatment of clinical syndrome (such as CNS disease) (28%), concern for alternative tickborne infection (3%), and no reason given (22%). There were no severe adverse reaction to doxycycline; 2 patients stopped due to nausea/vomiting and 1 due to refusal of oral medications. Among the 29 patients who completed doxycycline, there were no known Lyme disease treatment failures.

Presenting symptoms of children <8 years old treated with doxycycline for Lyme disease

**Figure:**
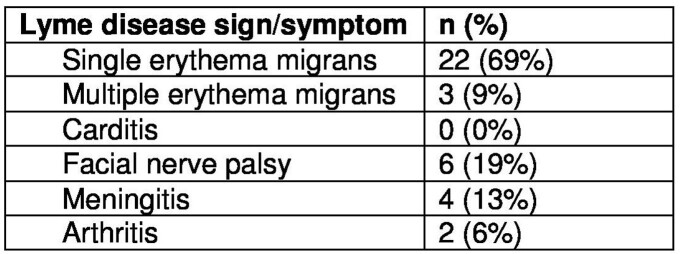

Total percent exceeds 100% due to patients presenting with multiple symptoms. Percent calculation based on all included patients (n = 32).

**Conclusion:**

In this small cohort of young children with Lyme disease, doxycycline was generally well-tolerated, without severe short-term adverse effects, and resulted in no treatment failures. As there is no prospective data evaluating doxycycline in this population and future randomized trials are unlikely, the data presented provides reassurance that doxycycline is effective and safe for this indication.

**Disclosures:**

**All Authors**: No reported disclosures.

